# Exploration of Genetic Variants within the Goat A-Kinase Anchoring Protein 12 (*AKAP12*) Gene and Their Effects on Growth Traits

**DOI:** 10.3390/ani11072090

**Published:** 2021-07-14

**Authors:** Yangyang Bai, Rongrong Yuan, Yunyun Luo, Zihong Kang, Haijing Zhu, Lei Qu, Xianyong Lan, Xiaoyue Song

**Affiliations:** 1 Shaanxi Provincial Engineering and Technology Research Center of Cashmere Goats, Yulin University, Yulin 719000, China; bai345@126.com (Y.B.); rongrong454250833@163.com (R.Y.); haijingzhu@yulinu.edu.cn (H.Z.); ylqulei@yulinu.edu.cn (L.Q.); 2 Key Laboratory of Animal Genetics, Breeding and Reproduction of Shaanxi Province, College of Animal Science and Technology, Northwest A&F University, Yangling 712100, China; lyy980909@126.com (Y.L.); amaranthkang@163.com (Z.K.); 3 Life Science Research Center, Yulin University, Yulin 719000, China; 4 Shaanxi Province “Four Subjects One Union” Sheep and Goat Engineering & Technology University & Enterprise Alliance Research Center, Yulin 719000, China

**Keywords:** goat, A-kinase anchoring protein 12 (*AKAP12*), insertion/deletion (indel), growth traits

## Abstract

**Simple Summary:**

*AKAP12*, the family of A-kinase anchoring proteins (AKAPs), plays an important role in the regulation of growth and development. There have been no corresponding studies of the effect of the *AKAP12* gene on growth traits in goats. In our previous study, 7 bp (intron 3) and 13 bp (3′UTR) indels within the *AKAP12* gene significantly influenced *AKAP12* gene expression. This study expected to identify the association between these two genetic variations and growth-related traits in 1405 Shaanbei white cashmere (SBWC) goats. The P1–7 bp indel locus was significantly correlated with height at hip cross (HHC; *p* < 0.05) and the P2–13 bp indel locus was associated with body weight, body length, chest depth, chest width, hip width, chest circumference and cannon (bone) circumference in SBWC goats (*p* < 0.05). These results prove that the *AKAP12* gene plays an important role in the growth and development of goats.

**Abstract:**

The A-kinase anchoring protein 12 gene (*AKAP12*) is a scaffold protein, which can target multiple signal transduction effectors, can promote mitosis and cytokinesis and plays an important role in the regulation of growth and development. In our previous study, P1–7 bp (intron 3) and P2–13 bp (3′UTR) indels within the *AKAP12* gene significantly influenced *AKAP12* gene expression. Therefore, this study aimed to identify the association between these two genetic variations and growth-related traits in Shaanbei white cashmere goats (SBWC) (n = 1405). Herein, we identified two non-linkage insertions/deletions (indels). Notably, we found that the P1–7 bp indel mutation was related to the height at hip cross (HHC; *p* < 0.05) and the P2–13 bp indel was associated with body weight, body length, chest depth, chest width, hip width, chest circumference and cannon (bone) circumference in SBWC goats (*p* < 0.05). Overall, the two indels’ mutations of *AKAP12* affected growth traits in goats. Compared to the P1–7 bp indel, the P2–13 bp indel is more suitable for the breeding of goat growth traits.

## 1. Introduction

The Shaanbei white cashmere (SBWC) goat is among the well-known breeds for both cashmere and meat in Northwest China. Today, the population of SBWC goats in Yulin is nearly 10 million. As important economic traits, breeders are concerned with growth traits [1]. The traditional breeding interval is long, and the selection intensity, selection efficiency and accuracy must be improved. Molecular marker-assisted selection (MAS) is among the most accurate and repaid methods which could satisfy the need to screen genes and consider the relationship between polymorphisms and growth-related traits. Herein, we propose that establishing an MAS system will speed up the development of goat breeding [2]. Insertion/deletion (indel) is characteristically widely distributed, highly polymorphic, stable and an easy to analyze [3]. It can be applied to the identification of functional genes that control traits, which is conducive to the further development and utilization of excellent genes, and is widely used in the fields of animal and plant population genetic analysis, molecular assisted breeding and human forensic genetics [4,5,6].

A-Kinase anchoring protein 12 (*AKAP12*) was initially identified in patients with myasthenia gravis [7] and was a known tumor suppressor [8,9,10]. *AKAP12* is a scaffold protein gene, which can target multiple signal transduction effectors, such as protein kinase A (PKA) and extracellular signal regulated kinase (ERK) [11]. Importantly, it is also plays a role in mitogenic regulatory activity and has a role in the control of both cell signaling and cytoskeletal arrangement. For example, Coats et al. (2000) highlighted that SSeCKS causes rat aortic smooth muscle cells (RASM) to interact with the intracellular signaling pathways that control cytoskeleton remodeling and extracellular matrix remodeling after Ang II stimulation [12]. In addition, in adult mice, A-kinase anchoring protein 12 shows the highest expression in smooth and cardiac muscle, indicating that *AKAP12* controls diverse developmental processes [13]. Kim et al. (2013) noticed that in the absence of *AKAP12*, zebrafish embryos had reduced locomotor activity; *AKAP12* is critical for the development of locomotor behavior in zebrafish through its regulation of muscle cell morphogenesis and migration [14]. In 2019, Messad found that the *AKAP12* gene is implicated in the regulation of cell development and muscle growth in pigs [15]. Furthermore, *AKAP12* is a vital gene to the cAMP signal pathway, the process of mammalian development and growth. For example, the bone morphogenetic protein (*BMP*) receptor family (*BMPs*) and growth differentiation factor 9 (*GDF9*) genes, which are crucial to the cAMP signal pathway, were all significantly associated with animal growth traits [16,17]. Overall, all the above results indicate that *AKAP12* plays an important role in the regulation of growth and development.

Our team discovered and determined 7 bp (intron 3) and 13 bp (3′UTR) indels in a previous study of the *AKAP12* gene, and constructed the expression profile of *AKAP12* gene in ruminants [18]. These two indel mutation sites probably change gene expression; first, because it is located in the 3′UTR region and can change the miRNA binding site, and second, it is an intron that can change the splicing of this gene or the binding sites of regulatory gene expression elements. Therefore, the purpose of this study was to explore and evaluate the effects of 7 bp and 13 bp indels on the growth traits of Shaanbei white cashmere goats. Thereby we also provide a theoretical basis for the application of molecular marker breeding in SBWC goats.

## 2. Materials and Methods

### 2.1. Ethics Statement

All animal tests performed in this study were conducted under the supervision and guidance of the Animal Welfare Committee of Northwestern Agricultural and Forestry University (NWAFU-314020038) and all procedures were in accordance with their specifications.

### 2.2. Animal Samples and Data Collection

Ear tissue samples from 1405 (2–3 years old) adult female Shaanbei white cashmere (SBWC) goats were selected. According to a family tree kept and recorded on the farm, there was no genetic relationship between individual goats. They were raised on a Shaanbei white cashmere goat farm in Shaanxi Province. All the goats were kept under standard conditions, including the same diet and feeding and management conditions [1,19]. The feeding programs were as follows: all the kids were continuously kept with their dams until weaning at the age of 3 months. Data on the growth traits of these goats, such as height at hip cross (HHC), chest width (CW), body weight (BW) body length (BL), chest depth (CD), hip width (HW), chest circumference (ChC) and cannon circumference (CC) were obtained.

### 2.3. Isolation of DNA

For these samples (n = 1405), Phenol-chloroform extraction method was used to extract genomic DNA from ear tissues [20,21]. The concentrations of 1405 samples were measured by a Nanodrop 2000 Spectrophotometer to assess DNA purity (A_260/280_ ratio) and quality, and were diluted to 10 ng/µL and frozen at −40 °C for further experiments.

### 2.4. Primer Design and Genotype Detection

P1–7 bp indel (NC_030816:g.83323del ACTGCTG, intron 3) and P2–13 bp indel (NC_ 030816.1: g.110266del TGGTCTTTTTGTG, 3′UTR) were detected in goats *AKAP12* [18]. A 13 µL reaction mixture and amplification steps (touch down-PCR) were undertaken as per to our previous studies [22]. PCR amplification was performed with an initial denaturation at 95 °C for 5 min, followed by 18 cycles at 94 °C for 30 s, 68 °C to 50 °C for 30 s and 72 °C for 12 s; then, 34 cycles at 94 °C for 30 s, 50 °C for 30 s and 72 °C for 12 s, with a final extension at 72 °C for 10 min were performed. PCR products were detected by Sanger sequencing and electrophoresis in agarose gel at 3.5% concentration [23,24].

### 2.5. Statistical Analysis

The Hardy–Weinberg equilibrium (HWE) of the *AKAP12* indels was examined using a chi-square (χ^2^) test. Nei’s method was used to calculate the genotype and allele frequencies [10]. The correlation between indels and growth traits was analyzed using a one-way ANOVA on SPSS software (version 24.0). Zhu’s methods were used to construct a linear model of the relationship between goat genotypes and each growth trait [25]. Statistical analysis showed that the age and birth season of goats had no significant influence on the growth of goats; thus, the age and birth season were not considered in the construction of the model.

### 2.6. Linkage Disequilibrium Analysis

Linkage disequilibrium (LD) analysis was performed on the P1–7 bp and P2–13 bp sites of genes using the SHEsis online platform (http://analysis.biox.cn/myAnalysis.php; accessed on 10 June 2021) [26]. The linkage degree (D’/r^2^) between the polymorphic loci was estimated as previously described [27]. In linkage disequilibrium analysis, the r^2^ value is preferred as an indication of the possible correlation between markers and the desired QTL, because it summarizes both recombination and mutation, and therefore represents a more statically accurate parameter when determining recombination differences. By contrast, when the sample size is too small, the actual meaning of the D’ value can easily be “exaggerated”, especially when the frequency of one of the alleles at a certain locus is very low [28].

## 3. Results

### 3.1. Indel Identification

**Figure 1:**
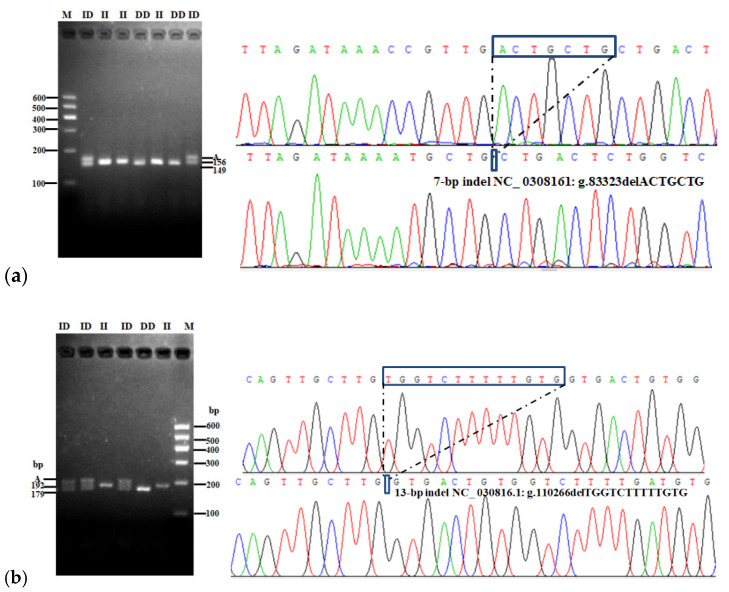
Agarose gel electrophoresis (3.0%) of PCR product of the goat *AKAP12* gene for P1–7 bp (**a**) and P2–13 bp (**b**) indel variants in Shaanbei white cashmere goats. Note: II, homozygous insertion/insertion genotype; DD, homozygous deletion/deletion genotype; ID, heterozygous insertion/deletion genotype. The M represents the marker. A represents the non-target fragment called heteroduplex.

Two indel loci were found to be polymorphic in SBWC goats and named P1–7 bp indel (NC_030816:g.83323del ACTGCTG, intron 3) and P2–13 bp indel (NC_ 030816.1: g.110266del TGGTCTTTTTGTG, 3′UTR) in the *AKAP12* goats, respectively. The P1–7 bp and P2–13 bp indels displayed three genotypes: II (insertion/insertion), ID (insertion/deletion) and DD (deletion/deletion) (Figure 1). DNA sequencing results showed that the P1–7 bp and P2–13 bp mutation loci of the *AKAP12* gene were polymorphic and could be detected by agarose gel electrophoresis and Sanger sequencing (Figure 1).

### 3.2. Analysis of Genetic Diversity

Allelic and genotypic frequencies were calculated for the two indels of *AKAP12* (Table 1). The amount of polymorphism information (*PIC*) is an important indicator of the degree of DNA mutation. *PIC* is divided into high polymorphism (*PIC* ≥ 0.5), moderate polymorphism (0.25 ≤ *PIC* ≤ 0.5) and low polymorphism (*PIC* ≤ 0.25). The *PIC* values of P2–13 bp (*PIC* = 0.210) and P1–7 bp (*PIC* = 0.265) in the Shaanbei white cashmere goats tested in this study showed low polymorphism and moderate polymorphism respectively. The genotypic frequency of the P1–7 bp and P2–13 bp indel loci did not correlate with the Hardy–Weinberg equilibrium (HWE) (χ^2^ test, *p* < 0.05). This disequilibrium could be attributed to the artificial selection.

### 3.3. Linkage Disequilibrium (LD) Analysis

**Figure 2:**
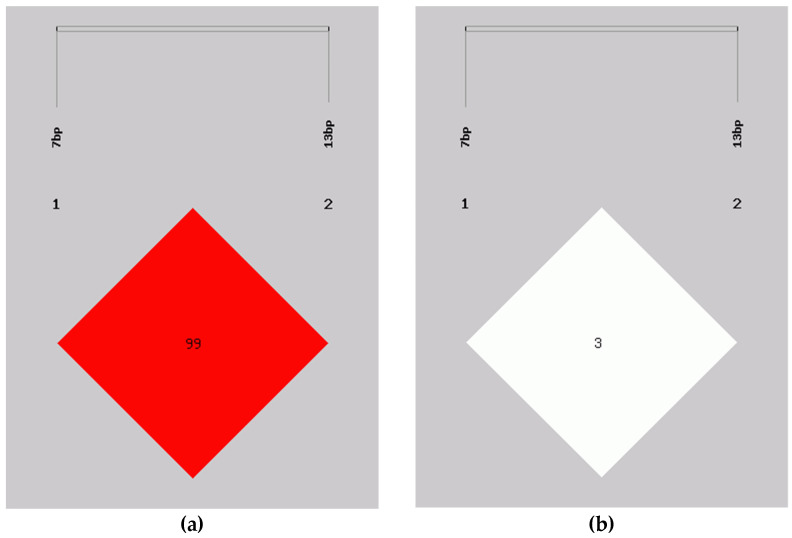
Linkage disequilibrium plot of the *AKAP12* gene two indel loci. (**a**) D’ = 0.997; (**b**) r^2^ = 0.031. Notes: “1, 2” represent the two mutation sites P1–7 bp and P2–13 bp of the *AKAP12* gene.

Based on the LD analysis results (Table 2; Figure 2), according to the D’ (D’ = 0.997) and r^2^ tests (r^2^ = 0.031) in the LD analysis, the P1–7 bp indel and the P2–13 bp indel loci were not closely linked in Shaanbei white cashmere goats.

### 3.4. Association Analysis of Indel Loci with Growth Traits in Goat

**Figure 3:**
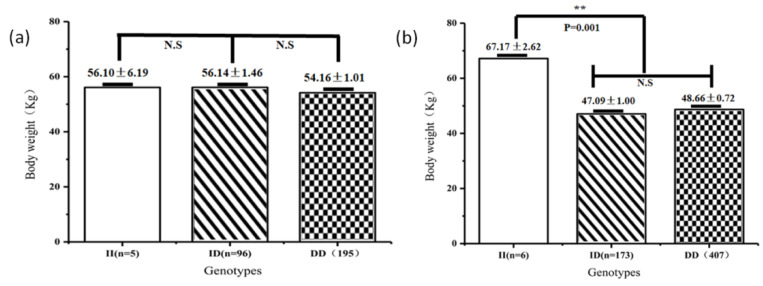
Association of the P1–7 bp (**a**) and P2–13 bp (**b**) indels with body weight in SWCG. Individuals with II genotypes had significantly (*p* = 0.002) higher body weight than ID and DD in the 13-bp indel of *AKAP12*. Data represents means ± SE. N·S means not significant; **: *p* < 0.01.

**Figure 4:**
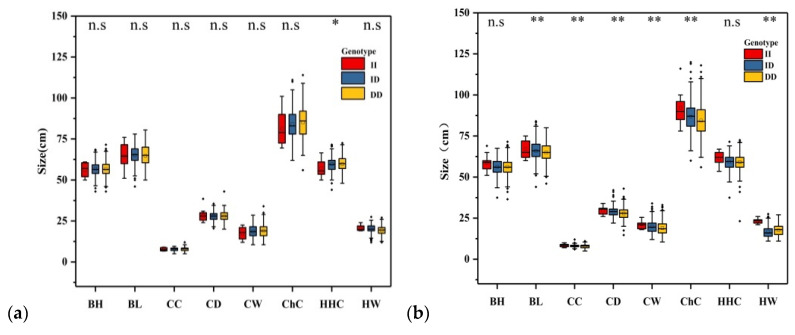
Relationship between P1–7 bp (**a**) and P2–13 bp (**b**) of the *AKAP12* gene and growth traits in SBWC goats. Note: BH, body height; BL, body length; HHC, height at hip cross; ChC, chest circumference; ChD, chest depth; ChW, chest width; CC, cannon circumference. Significance results refer to two test methods. N·S means not significant; * *p* < 0.05 and ** *p* < 0.01.

Table 3 shows the results of the correlations between the *AKAP12* indel loci and body measurements in SBWC goats. The effects of different genotypes on these traits varied. The P2–13 bp indel was highly correlated with body weight (BW; *p* = 0.001) (Figure 3), body length (BL; *p* = 0.005), chest depth (CD; *p* = 6 × 10^−6^), chest width (CW; *p* = 3.18 × 10^−4^), hip width (HW; *p* = 1.8 × 10^−5^) chest circumference (ChC; *p* = 1.32 × 10^−3^) and cannon circumference (CC; *p* = 0.007) (Figure 4). Individuals with II genotype were displayed relatively higher BW, BL, CD, CW, HW, ChC and CC compared with that of genotypes ID and DD. The P1–7 bp indel was related to height at hip cross (HHC; *p* = 0.013) (Figure 4), not associated with body weight (BW; *p* = 0.522) (Figure 3) and the individuals with genotype DD had higher breeding values for HHC.

## 4. Discussion

Breeding can make use of livestock resources and poultry breeds by playing the role of a precious gene bank of fine breeds, thereby improving the quality and quantity of livestock products [29]. In addition, it can also cultivate new varieties of strains, improve overall production performance, provide high-quality livestock products and maintain an competitive advantage in the market [10,30]. Goat breeding accounts for a very large proportion in the production of animal husbandry. Goat breeding accounts for a very large proportion in China’s animal husbandry production. As a dual-use species for fluff, Shaanbei white cashmere goats have the largest breeding stock in Shaanxi [31,32,33]. Therefore, improving goat production performance has an important role in increasing economic income. As one of the most important economic characteristics of goats, growth traits must be improved, as there is a current problem of slow growth rates that must be solved [34]. With the development of biotechnology, breeders have been choosing to use marker-assisted selection (MAS) in goat breeding. It is extremely important to improve the accuracy and predictability of the selection of superior genotypes for quantitative traits in the breeding process. To date, many quantitative trait loci (QTLs) affecting important economic traits in goats have been found [19,35].

Importantly, reproductive traits, like some of the complex quantitative traits, are polygenic, involving multiple genes and loci; we hope to find key genes for improving goat production performance [36]. In a previous study of myostatin (*MSTN*), it was found that it acts as key points during the pre- and post-natal life of amniotes that ultimately determine the overall muscle mass of animals. Bi et al. used a large population of goats to find that 5 bp insertion/deletion (indel) in the 5’untranslated region (5’ UTR) of the goat *MSTN* gene is associated with growth traits [34]. The growth differentiation factor 9 (*GDF9*) gene is a candidate gene for high prolificacy in livestock, and a novel 12-bp indel located within the *GDF9* gene significantly affected the growth traits [2]. This study hoped to explore the effects of two mutation sites in the *AKAP12* gene on growth traits in a large population of goats.

*AKAP12*, the family of A-kinase anchoring proteins (AKAPs), is a protein with the ability to regulate signal transduction processes. Cellular processes are regulated by *AKAP12* as a regulator of protein kinase A and protein kinase C signaling. *AKAP12* has been implicated in a wide range of cell functions, including cytoskeletal architecture [37] and cell cycle regulation. Previous studies have reported that the main role of *AKAP12’s* involvement in regulating different cell cycle stages is to promote cell mitosis and cytokinesis while acting as a negative regulator during inappropriate cell cycle progression [38]. As a scaffolding protein, *AKAP12* induces changes in cell shape and function during mesangial cell differentiation [39,40]. *AKAP12,* as a candidate gene, affects muscle development, and can affect a wide range of tissues and cell types through the downstream parts of the cAMP pathway, thereby regulating growth and development [15]. Previous studies have found that mutations of alleles of the *APAK12* gene were closely related to the growth and reproduction of embryonic cancer [41]. Based on these findings, we speculated that *AKAP12* was a candidate growth gene in goats.

To the best of our knowledge, there are no previous reports of goat *AKAP12* polymorphisms and their functional effects on growth traits in goats. According to our scan results, there are two indels (P1–7 bp and P2–13 bp) within the goat *AKAP12* gene. We took a large sample of 1405 SBWC goats as the research object, then used association analysis to explore the effects of the P1–7 bp and P2–13 bp indels of the *AKAP12* gene on growth traits. After electrophoresis and sequencing verification, each locus had three genotypes (II, ID and DD). The results showed that the mutation had the greatest effect on growth traits. In the analyzed sample, we found three haplotypes; hap1, hap2 and hap3, with frequencies of 0.680, 0.115 and 0.205, respectively (Table 2). In addition, LD analysis results showed that the P1–7 bp and P2–13 bp loci were not closely linked to the LD (D’ = 0.997, r^2^ = 0.031 respectively), suggesting that there was a minimal historical recombination between the two loci [18]. The relationship between these two loci of the *AKAP12* gene showed lower linkage disequilibrium, which is consistent with association analysis. The P1–7 bp and P2–13 bp loci were not correlated with the HWE (*p* < 0.05) due to the two mutations of *AKAP12*, the low frequency of allele I and the very low frequency of II genotype. Excessive and effective artificial selection is among the main reasons that the goat allelic of the indel locus do not correlate with the equilibrium. These two indels may be important genetic markers for goat breeding.

To analyze the association between indel loci and growth traits, we first used groups of 780 individuals, and only height at hip cross (HHC, *p* = 0.013) had a relationship with the P1–7 bp indel locus (*p* < 0.05). Interestingly, the P2–13 bp locus was consistently associated with body weight, body length, chest depth, chest width, hip width, chest circumference and cannon circumference in the same test groups (*p* < 0.05). Based on these data, we performed further analysis of the P1–13 bp indel among all individuals (1405) and found that the association with growth traits was retained (*p* < 0.05), with I alleles of the *AKAP12* gene positively affecting growth. In the process of raising goats, it is of considerable importance to select individuals with a fast growth rate and large body size to maintain the economic situation of the goat industry. In this study, for the P2–13 bp indel, Insertion/Insertion carriers showed better body weight and growth traits than deletion/deletion and Insertion/Deletion genotyped individuals in adult female goat populations. Although China has abundant goat breeding resources, poor growth and inferior quality still impede mutton production. From this perspective, the P2–13 bp indel may be suited to further selection and breeding.

To date, many regulatory elements have been described in introns [42]. Additionally, gene introns may contain cis-regulatory elements that participate in tissue- or stage-specific gene expression [28]. For instance, a novel intronic indel in the *HIAT1* gene has strong genetic effects on growth traits in goats [43]. A previous study [18] used RNA hybrids (http://bibiserv.techfak.uni-bielefeld.de/rnahybrid/ accessed on 19 January 2021) to predict miRNAs binding to the P2–13bp region. It was found that the miR–181 seed region could bind to the indel sequences. As we known, miRNAs can bind to the 3’-UTR of their target mRNAs to inhibit gene expression [44]. Therefore, we speculated that the P2–13 bp indel mutation might affect the goat growth traits by combining with miR–181.

## 5. Conclusions

The P1–7 bp and P2–13bp indels within the *AKAP12* gene were verified and were found to be significantly associated with the growth traits of SBWC goats via association analysis. Moreover, *AKAP12* could be regarded as an important genetic marker for goat breeding. Compared with the P1–7 bp indel, the P2–13 bp indel is more suitable for the breeding of goat growth traits.

## Figures and Tables

**Table 1:** Genetic parameters of two indel loci within *AKAP12* gene in Shaanbei white cashmere goats.

Loci	Size	Genotypic Frequencies			Alleles Frequencies		HWE	Population Parameters		
	N	II	ID	DD	I	D	*p* Value	*Ho*	*He*	*PIC*
P1–7 bp	780	0.018	0.355	0.627	0.196	0.804	0.00032	0.685	0.315	0.265
P2–13 bp	1405	0.9	0.258	0.733	0.138	0.862	0.002	0.762	0.238	0.210

HWE, Hardy–Weinberg equilibrium; Ho, homozygosity; He, heterozygosity; *PIC*, polymorphism information content.

**Table 2:** Haplotypic frequencies within the *AKAP12* gene in Shaanbei white cashmere goats.

Different Haplotypes	P1–7bp InDel—P2–13bp InDel	Haplotype Frequencies
hap1	D_7_D_13_	0.680
hap2	D_7_I_13_	0.115
hap3	I_7_D_13_	0.205
hap4	I_7_I_13_	0.000

“Hap” represents “haplotype”; indel: insertion/deletion.

**Table 3:** Associations of two indel loci within *AKAP12* gene growth parameters in Shaanbei white cashmere (SBWC) goats (mean ± SE).

Loci	Parameters	Genotypes			*p*-Values
		II	ID	DD	
P1–7 bp	BW (kg)	56.10 ± 6.19 (n = 5)	56.14 ± 1.46 (n = 96)	54.16 ± 1.01 (n = 195)	0.522
	BH (cm)	55.21 ± 1.42 (n = 14)	57.07 ± 0.28 (n = 277)	56.91 ± 0.19 (n = 489)	0.307
	HHC (cm)	56.05 ^ab^ ± 1.82 (n = 14)	60.09 ^b^ ± 0.29 (n = 277)	60.14 ^a^ ± 0.21 (n = 489)	0.013
	BL (cm)	63.82 ± 1.91 (n = 14)	65.41 ± 0.32 (n = 277)	65.08 ± 0.27 (n = 489)	0.506
	CD (cm)	28.37 ± 0.98 (n = 14)	27.71 ± 0.16 (n = 271)	27.82 ± 0.14 (n = 479)	0.659
	CW (cm)	17.61 ± 0.96 (n = 14)	18.63 ± 0.22 (n = 271)	19.20 ± 0.17 (n = 481)	0.053
	HW (cm)	20.08 ± 0.95 (n = 6)	19.88 ± 0.23 (n = 149)	19.43 ± 0.16 (n = 268)	0.248
	ChC (cm)	83.50 ± 3.02 (n = 13)	85.19 ± 0.60 (n = 0.60)	86.16 ± 0.45 (n = 488)	0.307
	CC (cm)	7.88 ± 0.27 (n = 13)	7.90 ± 0.57 (n = 278)	7.93 ± 0.45 (n = 489)	0.916
P2–13 bp	BW (kg)	67.17 ^A^ ± 2.62 (n = 6)	47.09 ^B^ ± 1.00 (n = 173)	48.66 ^B^ ± 0.72 (n = 407)	0.001
	BH (cm)	57.85 ± 1.40 (n = 13)	56.33 ± 0.22 (n = 362)	56.42 ± 0.15 (n = 1026)	0.504
	HHC (cm)	60.50 ± 1.32 (n = 13)	59.46 ± 0.24 (n = 361)	59.44 ± 0.15 (n = 1027)	0.724
	BL (cm)	68.31 ^AB^ ± 1.20 (n = 13)	66.02 ^A^ ± 0.31 (n = 362)	65.01 ^B^ ± 0.18 (n = 1027)	0.005
	CD (cm)	29.15 ^AB^ ± 0.62 (n = 13)	28.70 ^A^ ± 0.15 (n = 362)	27.86 ^B^ ± 0.96 (n = 1028)	6 × 10^−6^
	CW (cm)	21.42 ^A^ ± 0.65 (n = 13)	19.80 ^A^ ± 0.19 (n = 362)	18.94 ^B^ ± 0.12 (n = 1030)	3.18 × 10^−4^
	HW (cm)	22.63 ^A^ ± 0.75 (n = 8)	17.30 ^B^ ± 0.23 (n = 205)	17.90 ^B^ ± 0.15 (n = 491)	1.8 × 10^−5^
	ChC (cm)	92.77 ^AB^ ± 2.86 (n = 13)	87.21 ^A^ ± 0.48 (n = 358)	84.88 ^B^ ± 0.31 (n = 1027)	1.32 × 10^−3^
	CC (cm)	8.21 ^AB^ ± 0.24 (n = 13)	8.08 ^A^ ± 0.42 (n = 361)	7.92 ^B^ ± 0.31 (n = 1027)	0.007

BW, body weight; BH, body height; HHC, height at hip cross; BL, body length; CD, chest depth; CW, chest width; HW; hip width; ChC, chest circumference; CC, cannon circumference. Values with different letters (^a^, ^b^/^A^, ^B^) within the same row differ significantly at (*p* < 0.05/*p* < 0.01).

## Data Availability

Data are available upon request from corresponding author.
